# A multisectoral institutional arrangements approach to integrating civil registration, vital statistics, and identity management systems

**DOI:** 10.1186/s41043-019-0179-z

**Published:** 2019-10-18

**Authors:** Samuel Mills, Jane Kim Lee, Bahie Mary Rassekh

**Affiliations:** World Bank Group, 1818 H Street, NW, Washington DC, 20433 USA

**Keywords:** Civil registration, Vital statistics, Civil identification system, Institutional arrangements, Unique identification number, Policymakers

## Abstract

This paper reviews the essential components of a recommended institutional arrangements framework of integrated civil registration and vital statistics (CRVS) and civil identification systems. CRVS typically involves several ministries and institutions, including health institutions that notify the occurrence of births and deaths; the judicial system that records the occurrence of marriages, divorces, and adoptions; the national statistics office that produces vital statistics reports; and the civil registry, to name a few. Considering the many stakeholders and close collaborations involved, it is important to establish clear institutional arrangements—“the policies, practices and systems that allow for effective functioning of an organization or group” (United Nations Development Programme, Capacity development: a UNDP primer. New York: United Nations Development Programme, 2009). An example of a component of institutional arrangements is the establishment of a multisectoral national CRVS coordination committee consisting of representatives from key stakeholder groups that can facilitate participatory decision-making and continuous communication. Another important component of institutional arrangements is to create a linkage between CRVS and the national identity management system using unique identification numbers, enabling continuously updated vital events data to be accessible to the civil identification agency. By using birth registration in the civil registry to trigger the generation of a new identification and death registration to close it, this link accounts for the flow of people into and out of the identification management system. Expanding this data link to enable interoperability between different databases belonging to various ministries and agencies can enhance the efficiency of public and private services, save resources, and improve the quality of national statistics which are useful for monitoring the national development goals and the Sustainable Development Goals. Examples from countries that have successfully implemented the recommended components of an integrated CRVS and national identity management system are presented in the paper.

## Background

### Civil registration and civil identification systems

Sustainable Development Goal (SDG) Target 16.9 aims to provide legal identity for all, including birth registration, by 2030. To track the progress of this target, countries need to monitor the proportion of individuals who have a legal identity and the proportion of children under 5 years of age whose births have been registered with a civil authority [1]. The best sources of data for these indicators are national civil registration and vital statistics (CRVS) and civil identification systems of each country. Civil registration is defined as the “continuous, permanent, compulsory and universal recording of the occurrence and characteristics of vital events pertaining to the population, as provided through decree or regulation in accordance with the legal requirements in each country. Civil registration is carried out primarily for the purpose of establishing the documents provided for by law” [2]. It is a fundamental function of the national government. Among the ten vital events recognized by the United Nations (UN) [3], registering the occurrence and characteristics of a birth is the first step to establishing a legal identity and subsequently obtaining a proof of identity (for example, a birth certificate). A national civil identification system further adds attributes of the individual, such as a unique identification number (UIN) and biometrics (for example, a fingerprint, facial recognition, and an iris scan), which the individual can later use to prove legal identity.

When civil registration serves as a foundation for civil identification and the two systems are well-integrated, the UIN assigned to each individual at birth can serve as a common key to share data between the two systems and also with systems belonging to other institutions in both the public and private sectors. This linkage yields many benefits. First, this link accounts for the flow of people into and out of the identification management system. The birth registration with the civil registrar leads to the creation of an identity in the civil identification management system, and death registration with the civil registrar in turn closes the identity from the identification management system. Second, the linkage between the civil registration system and the civil identification system allows continuous updating of an individual’s vital events information throughout the person’s life cycle, from birth registration to death registration. For example, even if an individual changes his or her name, the individual’s UIN will stay the same and connect all vital events occurring in the person’s life. Third, UINs can facilitate the efficient exchange of reliable data between institutions for identity verification purposes, which ultimately results in improved public and private service delivery. One example is when an individual changes an address of residence. If an individual is required to report his or her change in residence to only one government agency and that triggers automated notifications regarding the address change to other government agencies and institutions that have the person’s UIN, such as financial institutions and tax agencies, then this process can benefit both parties. It can benefit the service providers by having information updated efficiently, and it can benefit the recipients by only having to report the address change once. This linkage between databases can also strengthen the quality and use of vital statistics—the statistics produced from data collected through civil registration—by creating a larger network of population data that can be used to (i) check the completeness and coverage rates of civil registration by comparing data on hand against other sources of data such as censuses, surveys, and hospital records; ii) perform more sophisticated data analyses that are useful for creating evidence-based policies and programs, hence contributing to good governance; and (iii) produce census-like small area statistics. The benefits of this model demonstrate clearly its efficiency and appropriateness in an increasing number of countries in which it has been implemented and is functioning [4].

### Institutional arrangements

Creating a system of institutional interoperability and collaboration and coordination of activities between several ministries and institutions that use civil registration and civil identification data requires close collaboration in their institutional arrangements. The United Nations Development Programme defines institutional arrangements as “the policies, practices and systems that allow for effective functioning of an organization or group. These may include ‘hard’ rules such as laws or the terms of a contract, or ‘soft’ rules like codes of conduct or generally accepted values” [5]. Legally binding laws, rules, or regulations can empower institutions to interact, and their absence may be a possible limitation to effective institutional arrangements.

**Fig. 1:**
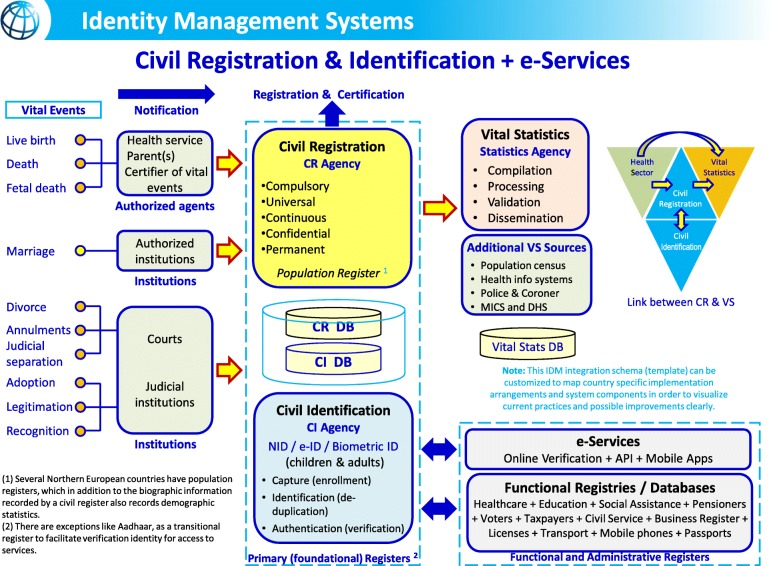
Integration of civil registration, vital statistics, and identity management systems. Source: World Bank Group Identification for Development (ID4D)

Aligning the policies, systems, and processes belonging to various organizations that work with civil registration and civil identification data involves several types of stakeholders. The stakeholders at the institutional level can be largely categorized into three groups: (i) the ministries/institutions that are directly involved with performing civil registration, producing vital statistics, and managing civil identification; (ii) the ministries/entities that use output from CRVS and civil identification as input to fulfill their mandate, such as the education sector that uses the number of births to plan the number of schools and teachers needed, and private businesses that use a prospective employee’s UIN and other proof of legal identity to verify work authorization; and (iii) the ministries/institutions that provide input into CRVS, such as the health sector that provides notification of new births and deaths (see Fig. 1), and the judicial system that provides information on marriage, divorce, adoption, among others. As such, wide-ranging ministries/institutions are intricately involved with various types of vital events, and thus, it requires that the institutional arrangements of CRVS and civil identification systems be multisectoral.

## Institutional arrangements framework

### National CRVS coordination committee with an anchor ministry

To effectively coordinate the collaboration among multiple stakeholders working with CRVS and civil identification, a coordinating mechanism at the national level is necessary. For this reason, the latest UN *Principles and Recommendations for a Vital Statistics System* [6] advises countries to establish an inter-agency coordination committee comprised of representatives from concerned ministries/institutions. This committee makes participatory decisions regarding strategic planning and implementation and oversees the major activities of the group such as the CRVS and civil identification system assessments, review of existing laws, development of standard operating procedures (SOPs), monitoring, and evaluation. To ensure smooth integration and continuous communication, it is recommended that the committee meets in person at least once per year.

Depending on each country’s context, the ministries and institutions that join the national coordination committee will vary, but some of the commonly participating ministries and institutions include the Ministry of Health, the Ministry of Home Affairs (or Interior), the Ministry of Foreign Affairs, the Ministry of Justice, the National Statistics Office, the Ministry of Education, and the Ministry of Gender/Child Protection, to list a few. Among the participating ministries and institutions, usually the ministry that oversees civil registration takes the role of an anchor ministry that serves as Secretariat to the national coordination committee, and that engages various stakeholders, including development partners, civil society organizations, and citizens, among others. A high-level official, such as the minister of the anchor ministry or a representative from the President’s or Prime Minister’s office or cabinet secretariat, is recommended to serve as the chair for the national coordination committee to strengthen political commitment and ensure convening power of that leadership. In Cambodia, the Minister of Interior serves as the chair for the national coordination committee, which meets every 6 months with representatives from eight line ministries to implement the national CRVS strategy [7]. In Bangladesh, the national CRVS steering committee is comprised of representatives from over 20 agencies and is led by the Cabinet Secretary [8].

### Integrating CRVS and civil identification systems using UINs

A conceptual framework that illustrates the link between civil registration, civil identification, vital statistics, and other institutions and administrative registries is shown in Fig. 1 [9]**.** Starting from the left, the ten vital events recognized by the UN are listed. When these vital events occur, often there are several institutions and authorities that are involved. For example, live birth, death, and fetal death are often attended by healthcare providers, making them ideal informants who can directly notify and provide pertinent information of the vital event to the civil registration authority. The same applies to the institutions responsible for authorizing marriage, divorce, adoption, and other events. Notification and transmittal of data involving vital events directly from these institutions to civil registration authorities in a way that provides the civil registry with all information required for the registration of the event can significantly increase the coverage of civil registration and lessen the burden on families. Operationalizing these collaborative efforts may include a number of actions including creating or amending CRVS-related decrees, regulations, or SOPs; creating a memorandum of understanding between ministries; and employing innovative methods such as using mobile phone text messaging for sending notifications. Co-location of health facilities and civil registration offices is another way to facilitate the sharing of pertinent information of vital events to the civil registration authority, which is particularly suitable for large hospitals. In Bolivia, through a partnership with the Supreme Electoral Tribunal and a private telecommunication company (TIGO), 25 in-hospital civil registration offices have been established. At the same time, collaborating with the education sector has allowed civil registration officers and education authorities to work together to identify undocumented students in schools to register their births [10].

Once registration information has been submitted to the civil registration authority, integration of data between the civil registration and civil identification systems is crucial to ensure that a registered person’s identity will be created and that the data will be uniquely identifiable and verifiable in the future. In Botswana, both the civil registration and national identification offices are housed within the same department under one ministry, while using a common UIN to link birth registration, civil identification, and death registration data [11]. In other countries, the civil registration and civil identification offices are not housed under the same department or the same ministry. Nevertheless, if the databases belonging to these two entities can be made interoperable using the UIN, then continuously updated vital events data can be accessible to the civil identification agency. The Netherlands has the civil registration and civil identification systems under two different ministries [12, 13]. While the Ministry of Justice and Security oversees birth registration, the Ministry of the Interior and Kingdom Relations assigns unique citizen service numbers and issues Dutch identity cards. In other countries, instead of governmental ministries, autonomous institutions have been given jurisdiction over civil registration and civil identification. The National Civil Registry and Identification Registry of Peru (RENIEC) provides an example of this case [14]. RENIEC is an autonomous entity (independent of any ministry) that is mandated by the constitution to provide integrated civil registration and civil identification services to its population. RENIEC also issues a unique code identification number (CUI) to each individual and includes this number on the birth certificate and national legal identity document so that individuals can use their CUI to access various services throughout their lifetime. As seen from the above examples, although the specifics of the institutional arrangements between the civil registration and civil identification systems may vary, using a UIN as a common key between the two systems has increased efficiency in many countries [15–17].

The use of UIN can also facilitate improving the quality of vital statistics. For example, if the authorities responsible for population censuses, health information systems, and health surveys can also collect UIN as part of their data collection and compare their data with CRVS agencies, then such assessments can provide a better picture of the completeness and coverage rates of civil registration and other surveys, resulting in higher quality national statistics. In some countries, such as in Norway [18] and Slovenia [19], a strong linkage between varied administrative registers makes it possible for those countries to produce a register-based census by compiling population information already collected in different administrative registers. This method is significantly more cost-effective compared to having to use multiple yearly population and housing censuses with enumeration forms [20]. It also reduces the response burden of the population, while enabling access to continuously updated population data instead of collecting it decennially.

Other administrative (functional) registers also benefit from having access to an integrated CRVS and civil identification system. For example, through continuous death registration data, the risk of fraud can decrease, such as in the case of the voter registry and service delivery systems which can ensure that individuals who have been reported as deceased are removed from the list in a more efficient manner. Similarly, cause-of-death data from death registration can inform the various disease registries, such as the cancer registry if someone has died from cancer, without doing extra data collection. In countries where UINs are assigned to individuals, a population register can link CRVS data and civil identification system data (primary or foundational registers) with data from other functional and administrative registers (e.g., registers for migration, health care, taxation, or schools) and thus create a broader network of up-to-date, reliable data pertaining to its population. The system is more cost-efficient and provides greater accessibility to the public when it is unified [21].

This type of data linkage and accessibility necessitates having in place appropriate laws and other measures that require all involved parties to maintain data confidentiality, privacy, and cyber security. Highly interlinked systems must ensure confidentiality of information; all systems need not have access to all data and data from all systems must not be centralized in one database, but rather linkable using the UIN. The UIN in particular, if not safeguarded, can make other personal information vulnerable. Thus, proper measures to ensure confidentiality of information must be put in place before such systems may be implemented securely.

Information and communications technology (ICT) solutions must also be able to support such interoperability and standards. Estonia offers a good example of an integrated population registration and identity management system that is connected to over 900 organizations and 170 databases in both public and private sectors through a data exchange platform called X-Road [16]. The endeavor took over 15 years, during which the government first assigned each citizen a personal identification code (PIC), upon which civil registration information was added to form a population register [22]. Then, national identification (ID) cards were issued, which are now also available as a digital ID card using mobile devices that can be used to access a myriad of public and private services. Both the population register and the national legal identity system are housed under the Ministry of Interior. It must be noted that X-Road, which is the data exchange platform supporting Estonia’s eGovernment system, does not copy and save all data in one database; rather, it inter-connects various databases using the PICs and allows these databases to share and reuse data. To reinforce the reuse of data, the Public Information Act [23] prohibits creating a new database and collecting information that is already available through X-Road. Operationally, public and private partnerships played an important role in advancing the undertaking. For example, to install Internet connectivity throughout the country, especially in rural areas, a private telephone company was involved. The government also worked with private ICT and iTech firms to build X-Road, to design the chips on the ID cards, and to print the ID cards. Among the many benefits of having this integrated system, one of the most utilized services is the e-prescription system in the health sector. Leveraging the inter-connection between the electronic health registry and electronic prescription system, all doctors in the country can view patient prescription history and put in new orders electronically, and patients are able to pick up their medicine from any pharmacy in the country. This process has significantly reduced physician time required for administrative tasks and has increased the convenience for the public, resulting in 99% of all medical prescriptions being serviced electronically. Another advantage of having this data-sharing in place is evident in the way health insurance is handled. In Estonia, all citizens are covered under the national compulsory social health insurance scheme, and the insurance database is updated nightly with information on new births registered in the population register. Therefore, it creates a strong incentive for parents to register the birth of their child immediately so the child can be included in the health insurance scheme.

## Conclusion

Civil registration and the provision of proof of identity is a fundamental function of a national government that establishes an individual’s legal identity and facilitates the realization of related rights and services. For the national government, having continuous and disaggregated data about its population and vital events available at all administrative levels enables targeted programs, services, and policies. Furthermore, statistics compiled from these data are essential for monitoring population trends and the progress toward achieving national development goals and SDGs. However, despite their crucial role in supporting good governance, CRVS systems in more than 100 low- and middle-income countries need substantial strengthening [24].

The institutional arrangements of CRVS and civil identification systems look different in different countries. Some countries manage both systems under one ministry while others have separate organizations. The types of agencies that are mandated with registering vital events are also diverse, from different government ministries to autonomous institutions. Nevertheless, due to the multisectoral nature of CRVS (that is, covering birth, death, marriage, divorce, adoption, and vital statistics, among others), maintaining effective coordination among the stakeholders is key to its successful implementation. For this reason, countries that are planning or are currently in the process of reforming their CRVS systems are highly encouraged to consider establishing a national, multisectoral CRVS coordination committee. This inter-ministerial committee can discuss and make participatory decisions regarding various aspects of CRVS, streamline processes and SOPs, and sustain and broaden institutional buy-in, among other benefits. Having an anchor ministry/agency that serves as secretariat of the national coordination committee is essential to ensuring the overall coordination and sustainability of the committee.

The benefits of CRVS can be multiplied when civil registration is linked with the civil identification system via a UIN. Information on vital events of individuals can serve as a reliable foundation upon which to build and connect other personal data collected by different ministries and institutions in both public and private sectors. Assigning a UIN at birth and using that same number for various transactions throughout a person’s lifetime makes it possible to link the data stored in different databases.

There are several countries that have taken steps in this direction, such as Botswana and Estonia, which were discussed earlier. The interoperability between databases supports the ability of these countries to have reliable legal identity authentication and efficient public and private service delivery, thereby contributing to strengthening governance.

## Data Availability

Not applicable.

## Abbreviations

CRVS: Civil registration and vital statistics
CUI: Code identification number
ICT: Information and communications technology
ID: Identification
ID4D: Identification for development
PIC: Personal identification code
RENIEC: National Civil Registry and Identification Registry of Peru
SDG: Sustainable Development Goals
SOP: Standard operating procedures
UIN: Unique identification number
UN: United Nations
